# Neurological recovery after early decompression for dorsal Pott’s spine

**DOI:** 10.1016/j.ijscr.2019.12.008

**Published:** 2019-12-13

**Authors:** Asayel Alruwaili, Muhammad Umerani, Amjad Darwish, Gabr Mostafa

**Affiliations:** a College of Medicine and Surgery, Imam Abdulrahman Bin Faisal University, Al-Khobar, Saudi Arabia; b King Fahd Military Medical Complex, Dhahran, Saudi Arabia

**Keywords:** Tuberculosis, Tuberculosis spondylitis, Pott’s disease, Kyphotic deformity, Anterior decompression

## Abstract

**Introduction:**

Tuberculosis spondylitis or Pott’s disease is the most common destructive form of skeletal tuberculosis. The most commonly affected site is the thoracolumbar vertebra. Once invading the adjacent structures of the vertebrae and intervertebral discs ultimately form an abscess causing spinal cord compression, vertebral collapse, and severe kyphotic deformity.

**Presentation of case:**

We present the first-ever case done in King Fahd Military Medical Complex in Dhahran, Saudi Arabia, of an 18-year-old male diagnosed with tuberculosis spondylitis who was complaining of progressive upper back pain and lower limb weakness. Emergent anterior decompression with expandable cage, plates, and screws were done. The patient showed a smooth and rapid recovery and was discharged with improved lower limb power and sensations.

**Discussion:**

Pott’s disease can lead to progressive neurological deficits in which surgical decompression is indicated. Current surgical practice includes anterior or posterior decompression with or without fusion and/or instrumentation.

**Conclusion:**

Surgery with either anterior or posterior decompression should assure complete clearance of the lesion, correcting the kyphotic deformity that prevents disease progression.

## Highlights

• Early evaluation of Tuberculosis spondylitis is necessary to decide for the appropriate management.
• Detailed medical history, meticulous examination, and prompt radiological investigation are important to reach for the right surgical decision.
• Early surgical decompression with correction of deformity prevents neurological deterioration and promotes adequate functional recovery.

## 1 Introduction

Tuberculosis spondylitis or Pott’s disease is one of the extrapulmonary manifestations of tuberculosis. Although uncommon in the western world, the reported cases are usually linked to immigrants from endemic areas and acquired immune deficiency syndrome [1,2]. While accounting for 50 % of the cases of extrapulmonary musculoskeletal tuberculosis, Pott’s disease affects about 1–2 % of tuberculosis cases worldwide [[1], [2], [3]]. Pott’s disease causes spinal complications by causing destruction of the vertebral endplates and intervertebral discs ultimately leading to spinal cord compression [1].

The diagnosis of Pott’s disease is usually made by laboratory, radiology, and histopathology. Magnetic resonance imaging with contrast is considered as the most appropriate diagnostic test especially at an earlier stage of the disease [1].

We report a case of an 18-year-old male diagnosed with spinal tuberculosis in the mid-thoracic region. Anterior right thoracotomy with D6 corpectomy with spinal cord decompression and fusion done by placing expandable cage, plates, and screws.

As per surgical case reports guidelines, this work was reported in line with the SCARE 2018 criteria [4].

## 2 Presentation of case

Our patient is an 18-year old male, patient originally from Jazan, Saudi Arabia, who was treated for pulmonary tuberculosis (TB) a few years back and had a positive family history of TB. He presented to the emergency department complaining of six months duration of progressive upper back pain with a one-month duration of lower limb numbness, associated with subjective fever, night sweats, and anorexia. This was associated with lower limb weakness, fecal and overflow urinary incontinence for approximately 5 days. The patient was not receiving any medications before the presentation as well as he denied the use of smoking and alcohol.

On examination he was lying uncomfortably on the couch, running low-grade temperature with stable blood pressure and pulse. He had been communicating well and was oriented. There was tenderness over midline at the dorsolumbar region where a gibbus was also noted. The patient had lower limb paraparesis with a lower limb power of approximately 1/5, restricted straight leg raising, positive ankle and knee clonus, bilateral +3 in knee jerk and plantar reflexes, negative Hoffman’s sign, lower limb hypoesthesia on light touch bilaterally up to inguinal ligament as well as loss of anal tone.

**Fig. 1:**
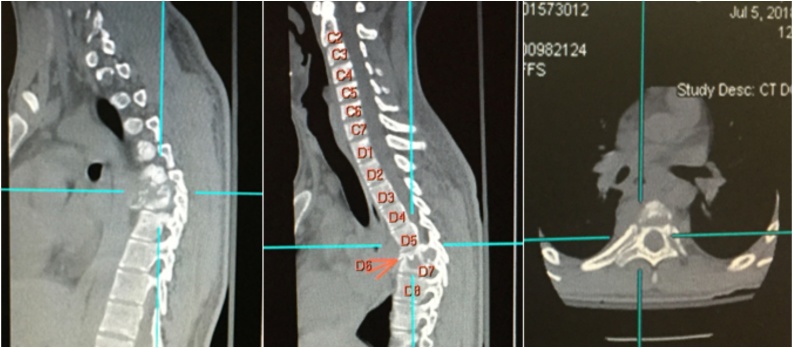
Pre-operative CT scan sagittal and axial images showing complete collapse of D6 and partial collapse of D7 vertebra along with kyphotic deformity.

**Fig. 2:**
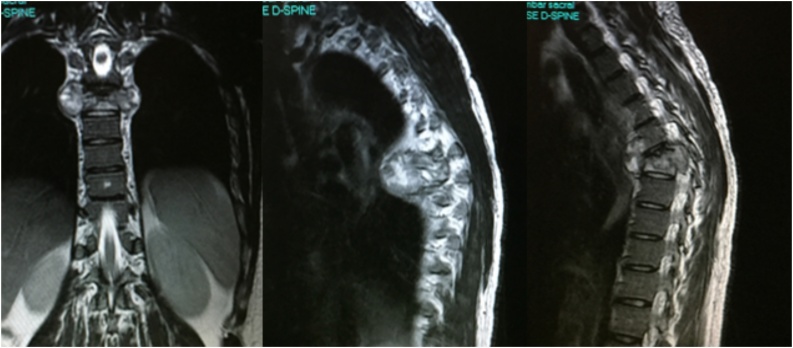
Pre-operative MRI (with contrast) scan coronal and axial images showing collapse of the D6 vertebra along with large pre and paravertebral abscess collection.

Initial Laboratory investigations showed erythrocyte sedimentation rate (ESR) of 53. Urgent computed tomography (CT) and Magnetic Resonance Image (MRI) of the dorsal spine showed completely collapsed D6 and partially collapsed D7 vertebrae with gibbus deformity surrounded by a large paravertebral abscess and anterior epidural abscess causing marked spinal cord compression, with possibly compressive myelopathy highly suggestive of tuberculosis spondylitis Figs. 1 and 2 .

Considering the abscess was mainly anterior to the spine, surgery was taken through the anterolateral approach through right-sided thoracotomy which was done by a thoracic surgeon. Intraoperatively, the granulation tissue and destructed bone were removed by the neurosurgeon, the epidural abscess was aspirated, and sent for histopathological detection of Tuberculum Bacilli. The dorsal cord was decompressed via D6 and D7 corpectomy, an expandable cage was placed and secured with plates and interbody screws. Thoracostomy tube connected to the underwater seal was placed that was removed on the 3rd post-operative day.

**Figs. 3 and 4:**
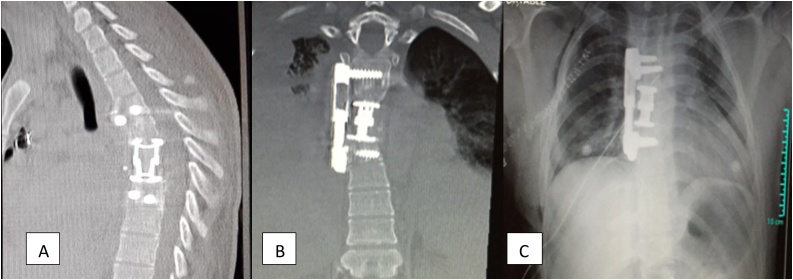
Post-operative CT scan coronal image (A & B) and chest X-ray image (C) showing fusion with cage and screw plate fixation.

There were no intraoperative or postoperative complications noted. Histopathology confirmed the diagnosis of tuberculosis spondylitis and anti-tuberculosis therapy was initiated. Postoperative CT scan and chest x-ray were done that showed restored spinal stability and corrected kyphotic deformity with a complete evacuation of granulation tissue. Figs. 3 and 4 On 3rd postoperative day, his power in the lower limbs started improving and later with the aid of physiotherapy, he started taking a few steps. The patient was satisfied as he underwent a successful recovery post-operatively. He was discharged on the 3rd week after surgery, ambulating via clutches with the power of 4/5 in lower limbs along with improved urinary and fecal incontinence, as well as lower limb sensations.

## 3 Discussion

Tuberculosis spondylitis is an uncommon disease, with an insidious onset and a slow progression [5]. Its incidence is high in countries with a high prevalence of pulmonary tuberculosis. The most commonly affected spinal site is the thoracic and thoracolumbar region [1,2]. While the presence of constitutional symptoms of weight loss, anorexia, and fever might be related to disseminated disease or concurrent extra-spinal (TB), severe back pain and neurological deficit occur due to worsening kyphotic deformity and neural compression respectively [1,6]. The disease progressively causes vertebral collapse, adjacent tissue damage, and destruction, eventually forming a paravertebral or epidural abscess. The abscess may extend to adjacent soft tissues, subsequently causing spinal cord compression, kyphotic deformity, and further neurological complications [2,7,8]. Our patient presented with a kyphotic deformity and progressive neurological deficits; therefore, an urgent surgical evacuation was recommended.

Surgical management in Pott’s disease depends upon the site of the lesion, extent of deformity and mass effect on adjacent neurological structures [2,9,10,11]. The clinical response to surgery is also faster and more complete in patients with active disease compared with those with chronic disease and deformity. Current surgical techniques are still controversial and include anterior decompression and fusion, with or without fixation; anterior decompression and fusion, with posterior fusion and instrumentation; posterior fusion with instrumentation and anterior decompression with fusion; posterior decompression and fusion with or without posterior instrumentation. These surgical approaches have been reported in the literature with several advantages and disadvantages as well as different outcomes and success rates [11,12].

Posterior decompression is favored for patients with neurological complications, kyphotic deformity, spinal instability, thoracolumbar and upper thoracic tuberculous spondylitis as a result of the complex anterior anatomical structures that might be injured during surgery [2,11,13].

However, anterior decompression is preferred in patients with extensive abscesses, mainly located in the anterior spinal column as it provides direct access to the vertebral body lesions. Therefore, global lesions, kyphosis, and instability are best treated with anterior decompression along with strut grafting and instrumentation. While anterior debridement followed by posterior instrumentation in the same surgical setting acquires a greater operative time, it dictates a better neurological outcome with acceptable spinal correction and a shorter hospitalization [12].

Thoracoscopic surgery, as well as percutaneous aspiration of the abscess, are recent minimally invasive techniques that can be considered in patients with a high risk of intraoperative neurological complications [8,13,14]. Since in our patient the abscess was extensive and mainly located anterior to the dorsal spinal column, we planned an urgent surgical decompression through the anterolateral thoracic approach.

## 4 Conclusion

The thoracic spine is the most common site affected in spinal TB. Early diagnosis and prompt treatment mandate good outcomes. The majority of patients are treated conservatively with anti- tuberculosis drugs. Only those cases who have global lesions, worsening kyphosis, and neurological instability require surgical intervention. Cases likely to develop severe kyphosis should be identified during the active disease & its correction should be attempted at the same time when the biological control is being achieved. Decompression with instrumentation, either anterior or posterior, in spinal TB is safe. This not only allows mechanical stabilization and prevents disease progression but also assists in early mobilization with a shorter hospital stay.

## Sources of funding

No source of funding.

## Ethical approval

Institutional Review Board approval. Case Report is presented anonymously.

## Consent

Patient is 18 years old and competent, we have written consent

## Author’s contribution

Asayel Alruwaili: Study concept, writing the paper, Communication, submission.

Muhammad Umerani: Writing the paper, Editing the paper,Study concept.

Amjad Darwish: Editing the paper.

Gabr Mostafa: Writing the paper.

## Registration of research studies

Not Applicable (Case Report; not an interventional study).

## Guarantor

Asayel Alruwaili.

## Provenance and peer review

Not commissioned, externally peer-reviewed.

## Declaration of Competing Interest

No conflict of interest to declare.
